# Navigating HIV research among criminalized gender minority populations in Uganda: qualitative insights and lessons learned from novice researchers

**DOI:** 10.1186/s12939-024-02294-1

**Published:** 2024-10-07

**Authors:** Patience A. Muwanguzi, Racheal Nabunya, Tom D. Ngabirano

**Affiliations:** School of Health Sciences, College of Health Sciences, Makerere University, Kampala, Uganda

**Keywords:** Criminalizing contexts, Gender minorities, HIV, LMICs, Qualitative study, Stigma, Sub-saharan Africa, Uganda

## Abstract

**Background:**

Transgender individuals often face stigma, discrimination, and various forms of abuse, which negatively impact their mental and physical health. They face a significantly greater risk of HIV, with a higher prevalence than the general population. Despite these challenges, transgender people have limited access to healthcare due to violence, legal barriers, and societal stigma, further exacerbated in countries like Uganda, where transgender identities are criminalized. Therefore, this study explored the lived experiences of HIV researchers working with gender minority populations in criminalizing contexts.

**Methods:**

This was an interpretative phenomenological analysis (IPA) qualitative study. Twelve (12) research team members at all levels were involved in the study. Participants had less than five years of involvement in HIV research among gender minority populations. Data were collected using field notes, reflective journals, documentation from daily team debriefing sessions, and semi-structured interviews. The analysis used NVivo software.

**Results:**

Positive experiences, barriers, and challenges were captured. The positive experiences were ‘respecting cultural diversity’, ‘expanding networks’, ‘addressing misconceptions’ and ‘finding allies’. The barriers included ‘experiencing stigma’, ‘lengthy research processes’, ‘feeling isolated’, ‘fearing for personal safety’, ‘unexpected logistical costs’, and ‘criminalization of sexual and gender minorities’. The key themes that emerged from the lessons learned were: ‘dealing with gatekeepers’, ‘diversity and sensitivity training’, ‘leveraging networks’, ’meaningful community engagement’, ‘reflexivity’, ‘ensuring safety’, ‘equal partnership’, ‘giving feedback’ and ‘awareness of legal implications’.

**Conclusions:**

This study highlights the importance of cultural sensitivity, community engagement, and reflexivity in research design and implementation. The findings emphasize the need for innovative strategies to navigate legal, social, and logistical barriers that researchers and participants face. Despite these challenges, the study demonstrates that meaningful collaboration with community members and building trust can significantly enhance the research process and outcomes. Future research should continue to explore these strategies while addressing ethical and safety concerns.

## Background

People who identify as transgender have gender identities, gender expressions, or behaviours that differ from those typically associated with the sex to which they were assigned at birth [1]. They frequently experience stigma, discrimination, and poor treatment because of their presumptive sexual orientation and gender identity or gender expression [2], which negatively impacts their mental and physical health. Furthermore, transgender people face various forms of gender-based abuse from a wide range of perpetrators, including romantic partners, family members, friends, members of the law enforcement community, and medical professionals [3, 4]. They are also at risk of acquiring HIV because of stigma and gender-based violence (GBV), which prevents them from using HIV prevention, care, and treatment services [3].

According to the World Health Organization (WHO), transgender people are about 49 times more likely to acquire HIV than other adults of reproductive age [5]. Additionally, the HIV prevalence among transgender women in Eastern and Southern Africa is estimated to be 28.4% [5]. Furthermore, one in every five transgender people in Kampala, Uganda’s capital, tested positive for HIV [6]. They also engage in risky behaviours such as coerced sex, inconsistent condom use, multiple sexual partners, alcohol consumption, and receptive anal sex with men [6]. This highlights the critical need for transgender HIV prevention and treatment programs in Uganda. Addressing the underlying social and cultural factors that contribute to their vulnerability to HIV is critical.

Despite the evidence presented above, transgender people have low rates of access to healthcare [7–11] due to a variety of issues, such as violence, legal barriers, stigma, and discrimination [5, 6]. Furthermore, they have a low education level and are unemployed, making it difficult for them to negotiate for better, non-stigmatizing healthcare services [12]. Despite this, transgender people are negatively impacted in countries like Uganda, where being transgender is prohibited and criminalized [13]. For example, the Ugandan parliament passed an anti-homosexuality bill on March 21, 2023. The proposed legislation makes it a crime even to identify as lesbian, gay, bisexual, transgender, or queer (LGBTQ+) and imposes harsh sentences, including the death penalty for “aggravated” homosexuality, which includes sex when the “offender” is an HIV-positive person [14].

Such actions impede sexually transmitted infections (STI) and HIV/AIDS prevention and control interventions among this minority group, threatening to reverse the country’s HIV response progress. Consequently, HIV researchers from academic institutions and non-governmental organisations conduct research among transgender people to identify such challenges and inform policy formulation. However, they frequently face methodological challenges during research [15]. These difficulties include identifying the correct gender, participant reluctance to answer questions about their gender, mistrust, and stigma [16, 17]. Furthermore, because it is a minority group, conducting the study is labour-intensive and costly to recruit an adequate sample size of participants [18].

As a result, phenomena such as HIV prevalence, gender-based violence, and STIs among transgender persons go unreported, hampering efforts to improve transgender welfare and HIV control. This information gap impedes the development of effective interventions and policies to address the specific needs of transgender people living with HIV in these areas, perpetuating health disparities and inequalities. Therefore, it is vital to prioritise research and advocacy efforts to increase awareness and understanding of the challenges faced by transgender persons in accessing HIV prevention, treatment, and care services in Sub-Saharan Africa. This study was positioned in that lacuna. We explored the lived experiences of HIV researchers who work with transgender people in hostile and oppressive environments. The central research question was: *What challenges and insights do novice researchers encounter while conducting HIV research among criminalized gender minority populations in Uganda*,* and how can these experiences inform future studies?*

## Methods

### Study design

The interpretative phenomenological analysis (IPA) method was used in this study. IPA is a qualitative research approach investigating how people make sense of their lived experiences [19]. It attempts to explore personal experience and is concerned with an individual’s perception or account of an object or event, as opposed to an attempt to produce an objective statement of the object or event itself [20]. The central question was, ‘What *are the experiences of HIV researchers working with gender minorities in highly stigmatising and criminalising environments?*’.

### Study participants

Participants were initially selected by purposive sampling and then by snowball sampling. They included research assistants, administrators, trainers, data entrants, manuscript authors, project directors, and principal investigators who consented to share their experiences.

We reviewed existing published literature to identify researchers studying any aspect of HIV among selected key populations, including men who have sex with men, sex workers, and transgender people. We then contacted the corresponding author of each publication to determine whether they had been working with transgender and other gender-diverse populations for five years or less. We requested the phone numbers and email addresses of various members of the research teams who met the criteria. We asked to meet each member individually or as part of a group. On the agreed dates, we visited the active project sites and presented the details of their study. Members interested in participating received additional information privately and consented to an interview after being fully informed. Participants without active projects selected a preferred venue for their interview or met us at our project site. Participants outside Kampala district participated in virtual interviews conducted via Zoom.

None of the participants self-identified as belonging to a gender minority population.

### Data collection

Data were collected using field notes [21], reflective journals [22], and documentation from daily team debriefing sessions, interviews, and focus group discussions. The authors (PAM and RN), a research assistant, and a social scientist gathered the data. They have received gender diversity and sensitisation training and have prior experience with qualitative methodologies.

#### ***Key informant interviews***

Two research team members with prior experience in qualitative research conducted the semi-structured key informant interviews (KIIs). The investigator had a list of questions on an interview schedule, but the discussion was guided rather than dictated by the schedule. This process allowed the researcher and participant to engage in a dialogue where the investigator could probe vital areas from the participant’s responses to the initial questions. The interview guide was iteratively tested and developed during two pilot interviews to improve the questions. The pilot interviewees consented before participating.

During the interviews, we used a semi-structured approach with open-ended questions, with room for probing, following an interview guide to gain deeper insights into their experiences and opinions. The guide elicited participants’ information about their experiences working with gender minorities in stigmatised environments. The interviews lasted about an hour each and took place between June and August 2023. We sought consent to audio record the interviews and take notes. Data saturation guided the sample size.

#### ***Document review***

We accessed field notes, researchers’ reflective journals, and documentation from daily debrief sessions. We reviewed the texts after the documents’ owner and the study’s principal investigators consented. We asked critical questions of the text, such as, “*What is the person trying to achieve here? What did they think when they wrote this? Is it my perspective that I’m bringing into this*,* or am I reading this text exactly as the participants intended?”*

### Data analysis

The interview recordings, participants’ field notes, and reflective journal entries were typed verbatim, and each de-briefing meeting’s recording was transcribed verbatim.

The analysis aimed to try to understand the content and complexity of those meanings rather than measure their frequency [19]. The author and two data coders read the transcripts more than once to understand the story. In the left margin, the coders wrote down interesting or significant parts of the respondent’s words; in the right margin, they wrote down the titles of emerging themes. In the second stage, the order was more analytical. They reviewed the transcription to ensure that the grouping of themes made sense and fit the participants’ words. The coding team eliminated certain themes that didn’t fit well with the new structure or needed more evidence to back them up.

The four trustworthiness criteria guided the study to ensure the process’s rigour [23]. Methods such as instant transcription, triangulation, and saturation established the findings’ credibility [24]. Four participants reviewed the transcripts and confirmed the final categories. Transcripts and recordings were used to create an audit trail presenting the dependability procedures and methods. Thick descriptions will allow the reader to decide how to apply the information to their contexts for transferability. We used field notes to establish confirmability.

## Results

### Lived experiences of conducting HIV research among gender minority populations

**Fig. 1:**
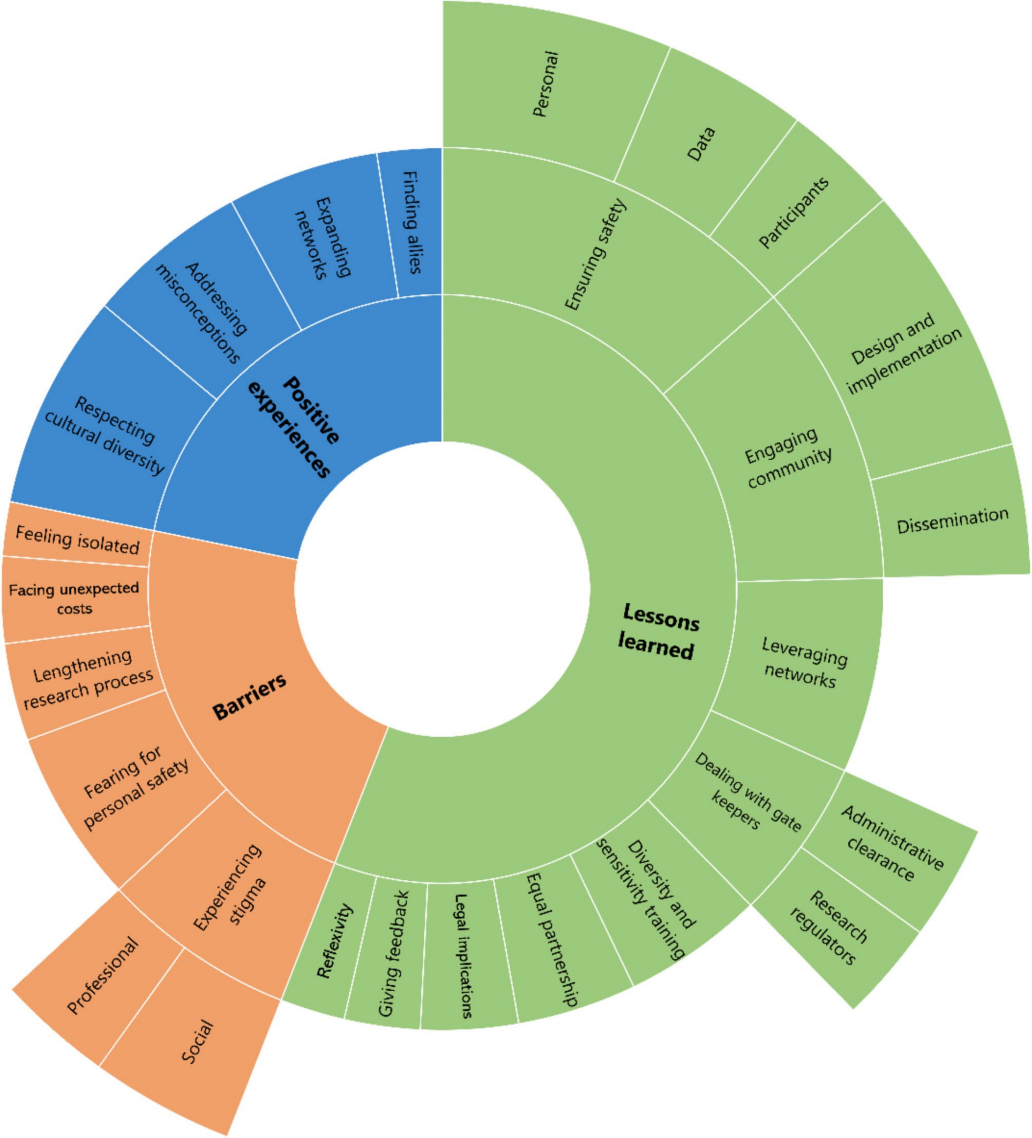
Coding tree for the participants’ lived experiences of conducting HIV research among gender minority populations

Three themes emerged from the participants’ lived experiences of conducting HIV research among gender minority populations. The themes, categories, and sub-categories are presented in Fig. 1.

All the narrative quotes are presented in Table 1.

**Table 1:** Narrative quotes of participants’ experiences of conducting HIV research among gender minority populations in Uganda

Theme		Quote #		Quote		KII #	
Positive experiences							
Respecting cultural diversity		1		“It was like learning a new language, unfamiliar words, and new expressions that I did not even know existed. Initially, the participants did not correct us, possibly out of fear…. But later, as trust grew, they helped us learn some things, like using correct pronouns.”		“”12	
		2		“This experience was a revelation for me. It was so refreshing to learn new things in a safe space for us and further research participants. I remember one peer educator called it an exchange of knowledge. She said …teach me about HIV, *and I teach you about being transgender*. ”		4	
Expanding networks		3		“Before beginning this study, I was determined to improve transgender people’s access to HIV services. I knew they existed, but I had no idea where or how to locate them. I will always be grateful to the lady who introduced me to the first transgender-led organisation because that organisation connected me to another organisation. Eventually, I was able to be part of a network.”		8	
		4		“The participants found participating much easier when we worked with their leaders. I realised there is a whole network and healthcare system that can be used to design HIV interventions for marginalised and ‘hidden’ populations.”		3	
Addressing misconceptions		5		“It is sad that we make decisions about people without facts. Working with this population cleared the air on many issues they had, and these were misconceptions that mainly came from misinformation on social media and movies.”		2	
		6		“because of this, we agreed to write reflective journals daily as a team. This helped us acknowledge our biases and assumptions and bracket them to ensure they did not colour our interpretation of research findings. Some team members did not want to meet the study participants but were comfortable working with the data.”		10	
Finding allies		7		“even in hostile environments like ours, you will always find partners and people fighting for human rights and justice and the healthcare of all members of society. This was surprising given the nature of the support we found, including [*removed for confidentiality*]. This was encouraging to me because it meant we could figure out a way for these populations to get HIV care, which is our goal. As a result, researchers should look in unexpected places to find people who will support them.”		1	
Negative experiences							
Experiencing stigma and safety implications		8		“This was one of the worst experiences. I was constantly bullied at work, and every time, people were asking me if I was gay. My colleague started calling me the ‘*transgender woman’*. ”		6	
		9		“I was surprised because this was coming from fellow HIV health providers who should know better since key populations are crucial in the fight to end the HIV epidemic. To me, this is just a lack of knowledge and understanding. Training can solve this.”		1	
		10		“I was constantly afraid…. I didn’t tell anyone about it…. I couldn’t discuss it with my family. I was always worried about the threats and the possibility of either having my premises raided or even being jailed for working with a population that was considered illegal.”		9	
		11		“I think for the safety of the researchers and participants, the concerned government bodies should put some guidelines and protocols that protect us while conducting research. These research findings are not for me; they are meant to improve the healthcare of Ugandans and to help develop policies.”		11	
Feeling isolated		12		“I found this work very lonely, and most of the time, I did it alone because I wasn’t sure who would be willing to discuss sexual and gender minorities. This became worse after passing this anti-gay bill; people did not even want to be seen talking to me for fear of being labelled sympathiser or recruiter, which seems to be the greatest crime. I have found a few other people working in the same area, so it’s much better now.”		7	
		13		“I know we have safe spaces for LGBT communities; this is part of what I learned during this research, but I think it would be nice to have welcoming and inclusive environments where researchers and health workers can come and talk openly about sensitive topics. It’s only in discussing these things openly that we shall overcome this stigma and improve access to healthcare.”		6	
Discrimination and fear for the safety of participants and researchers		14		“I don’t know if I was being paranoid, but suddenly, I had to be very careful about what I said and posted on social media……. I just felt watched. I had collected some interesting data but couldn’t even consider publishing it. ”		8	
		15		“Since the passing of this anti-homosexuality bill, I have changed from working with gender minorities I have changed from working with gender minorities I’m now working with adolescent girls and young women; that is a safe topic……” ***Probe***: *so*,* what do you think will happen to your unfinished research among trans people?* “maybe some other researchers who are stronger than me can continue that work.”		4	
		16		“Because of stigma and discrimination, we cannot meet our participants in public places other than hospitals. They must be met in safe areas where neither they nor the researcher is at risk. These venues are usually costly, so we must pay for them.”		1	
Lengthy research process and ethical considerations		17		“I applied simultaneously with four other colleagues, and they received ethical approval within a couple of months, but mine took seven months. Some of the meetings I spent answering questions about gender dysphoria, whether being transgender was legal in Uganda, and questions that were in no way related to my methodology or protecting human subjects.”		5	
		18		“I was very bitter because the funding period was just one year, and by the time I received approval seven months later, I only had five months remaining, and that was not enough time to conduct the study. It felt that some of the reviewers’ requirements were intentionally unrealistic and deterrent.”		5	
Unexpected logistical costs and obstacles		19		“in my first year of working with transgender women and gender non-binary people, I had to divert so many budget lines to participant compensation. I had grossly under-budgeted. Eventually, I had a limited budget for other parts of the study and sought additional funding support. Since then, I have worked with members from the trans community during budgeting, which prevents me from having unexpected expenditures during the implementation phase.”		9	
(Expectations of money)		20		“People believe that conducting research among these populations is for financial gain. As a result, we receive a barrage of requests for funding assistance, school fees from communities, and money for food to support the communities’ livelihoods.”		7	
Criminalization of sexual and gender minorities and implications for researchers		21		“Since it was passed, some participants have not taken my calls, while others have changed numbers. We had recently started recruiting for a clinical trial, but the enrollment numbers have decreased, and the few we recruited did not attend the first follow-up visit. ……Yes, all of them, which was suspicious but understandable.”		10	
		22		“I am still at war with myself…. As a health researcher, on which side of this anti-homosexuality legislation am I? Does inviting people to the health facility to access HIV services and battle my study amount to recruitment? How can I ensure that my participants continue receiving HIV prevention and HIV treatment? How are my research participants protected? What would my role be if anything happened and they were imprisoned for participating in this study? These are the kinds of tough questions I battle with daily.”		8	
Lessons learned							
Navigating the ethical approval and study site engagement process (Research regulation)		23		“I learned the value of community. In the next ethical approval application, I spoke to a colleague who had been doing research among men who have sex with men for about 10 years. I found this guidance and support very helpful. I think that by talking to other HIV researchers who work with similar populations and have more experience, people can learn about potential problems and how to deal with them.”		2	
(Administrative clearance)		24		“I wish I knew better…… but now I know that before even starting the study, I need to go to the different stakeholders and the people who have power in the areas where I plan to collect this data. This early engagement signifies respect and makes gaining entry, approval, and stakeholder buy-in easier.”		12	
Diversity and sensitivity training		25		“Since I had no experience, the Ethics Committee sent me to mandatory training on diversity and sensitivity. It was the best choice, and I recommend that any researcher who wants to study sexual or gender minorities for the first time go to a training like this if one is offered. We had never collaborated with a trans person before, and talking with the peer trainers, hearing their stories, and learning more about transgender healthcare gave me more confidence. The training also gave me the information I would not have had otherwise.		4	
Leveraging networks through trust building		26		“I wish I could change things about my first year, but I cannot. I used all my network connections to recruit participants and get more information I could use in other studies. I should have found trans experts and scientists from these communities and collaborated to plan and carry out future studies. After trying this method for about three years, I tried it, and the rest is history. So it’s important to use these networks, but make sure you do it for the right reasons and put the needs of people from gender minority groups first.”		11	
Meaningful community engagement and participation (Design and implementation)		27		“All I can say is that if you want your project to succeed, be effective and be sustainable, just involve members of the population at all stages of HIV research, from the study’s conceptualization to the protocol design to the pursuit of ethical approval, to the implementation of their study……no…no…. don’t just involve them, listen to them, listen to their concerns after all these are their realities, so they’re the experts on what they go through especially for us who are not members of that population”		3	
(Dissemination)		28		“I made a joint presentation with one of my partners from a transgender-led organisation I turn HIV conference. ………. people had a chance to interact directly with a community member who faced the challenge and was now reporting about the intervention and how it had changed their life. This approach also helped us to build trust and mutual respect with our participants.”		6	
		29		“……. involving community members in the dissemination process provides immediate feedback and increases the acceptability of the research findings.”		11	
Reflexivity		30		“I tried extremely hard but could not change my attitude towards these populations. I spoke to the PI about it, and he introduced reflexive channels where we had to write how we felt at the end of each day, where we stood in terms of our positionality, and how we thought this was affecting our work. This was very helpful for me because I also learned something about myself and my biases.”		12	
		31		“yes, I remember the journals…. I know it started out as writing about our biases, but some days were so hard, especially when I saw the stigma and discrimination that transgender people were facing, even from some members of the research team. Writing in this journal became therapeutic as a coping mechanism.”		9	
Ensuring safety (Personal safety)		32		“The discussions around this anti-gay bill were so many and so worrisome. When we received information that one of our participants had been assaulted, we decided to halt the study.”		6	
(Participants’ safety)		33		“We revisited our risk management and safety plan because there was now a real danger for our participants. We also sought approval to change the mode of data collection, making it more flexible.”		12	
(Data safety)		34		“The data safety plan needed strengthening beyond just identifying participants’ data; we needed to think about storage, particularly electronic data. I would recommend that researchers working with these populations write up a clear and explicit data safety plan. Such a plan should also include measures to ensure that the data is only accessible to authorized personnel and that any breaches are reported immediately to the affected individuals and appropriate authorities.”		7	
Equal partnership		35		“The transgender individuals are not your typical research participants; they are interested in participating in the process.”		3	
Giving feedback		36		“The participants often complained at the start that we conducted this research, but they did not get feedback that was specific for them. We now organise specific dissemination sessions where we invite the leaders of the gender minority groups and community members and present the findings. It is an open and free session to encourage dialogue.”		7	
Knowledge of legal implications		37		“I began my research career with gender minority populations two years ago and sought the legal services of one of the organisations that provide legal aid to LGBT populations. I took it a step further and earned a certificate in gender and human rights, which has come in handy in the current climate. To work with these populations, I recommend being fully informed about the legal status of trans people, prevailing legislation that affects transgender people structurally, and the consequences of breaking the law. I also recommend locating an excellent legal aid clinic or representation knowledgeable in these human rights issues.”		10	

### Positive experiences

The participants reported positive experiences while conducting HIV research among gender minority populations.

#### Respecting cultural diversity

Several participants reported that their interactions with gender minority populations gave them a new perspective on the world. They learned to respect cultural diversity by learning about gender identity and expression. Specifically, this included topics that the trans and gender non-binary people were willing to discuss, such as their sexual orientation and the gender identity of their partners. It was like learning a new language or learning something new about a completely different culture for several of them, which several participants appreciated (Quote #1). They also admitted to making and learning from many mistakes at the outset, such as misgendering their participants and focusing solely on the HIV research aspect of interest rather than looking at the participants as a whole person (Quote #2).

#### Expanding networks

Several researchers highlighted the new networks formed due to interactions with gender minority populations. This was an opportunity for several researchers to leverage existing networks, such as transgender-led organisations, drop-in centres, and health facilities that provide care to sexual and gender minority people (Quote #3). Through these networks, the researchers gathered data and insights on the unique health needs and experiences of transgender individuals (Quote #4).

#### Addressing misconceptions

Before beginning research with gender minority populations, several participants stated they had underlying negative prejudices. They reported that many of these misconceptions were addressed by interacting and engaging with members of these communities (Quote #5). They realised that much misinformation exists about these populations. The team also acknowledged that their biases and assumptions contributed to these misconceptions. They were committed to continuing their efforts to learn from and work with these communities respectfully and collaboratively (Quote #6).

#### Finding allies

Several participants discovered that, despite the stigmatising and criminalizing environment, some allies in various locations could provide support, guidance, resources, and information about HIV care for gender minority populations. This finding also meant that if people wanted to develop HIV interventions for gender minority populations, there were places where they could begin and be successful (Quote #7).

### Negative experiences while conducting HIV research among gender minority populations

The participants also reported several barriers to conducting HIV research among gender minority populations. The coding tree is provided in Fig. 1, and the narrative quotes are in supplemental Table 1.

#### Experiencing stigma and safety implications

##### Professional

Participants reported stigmatising professional experiences, such as bullying at work and being labelled homosexual or gay (Quote #8). This surprised them, coming from other HIV health professionals, primarily since key populations are known to have the highest risk of HIV transmission and acquisition. They felt that these experiences highlighted a gap in understanding and the need to address stigma within the HIV research community to prevent negative consequences for key populations (Quote #9).

##### Social

Some participants reported that because working with transgender people and other gender-diverse populations is criminalised, they did not even tell their immediate family that they were conducting health research among these populations. They expressed concern that they could not entrust this information to anyone because they feared workplace or home raids for their safety, incarceration, or even death due to community violence (Quote #10). They proposed that policies and legal frameworks that protect the rights of gender-diverse populations and those who work with them and ensure their safety and security are implemented immediately (Quote #11).

#### Feeling isolated

Some participants reported feeling isolated due to the negative social connotations associated with working with these populations. They thought they could not discuss their work or findings with anyone because they did not know what other people thought about sexual and gender minority populations (Quote #12). They emphasized the significance of creating a welcoming and inclusive environment for researchers on sensitive topics (Quote #13).

#### Discrimination and fear for the safety of participants and researchers

One of the most traumatic experiences mentioned by several HIV researchers was living in constant fear for one’s safety. Several researchers reported intense scrutiny of what they published and said, particularly on their social media platforms (Quote #14). Following the passage of the anti-homosexuality bill, some decided to focus on HIV care and prevention in other populations rather than sexual and gender minority populations for their safety (Quote #15).

The researchers were also responsible for the participants’ safety, ensuring they were not put at any additional risk due to their involvement in the research. “*Because of stigma and discrimination*,* we cannot meet our participants in public places other than hospitals. They must be met in safe areas where neither they nor the researcher is at risk.* (Quote #16).

#### Lengthy research process and ethical considerations

Because of their research topic, some researchers felt that the research process and required permissions to conduct the research could have been shorter. For some, regulators and other powers made unrealistic requests that took so long for the researchers to fulfil, causing delays (Quote #17). Some of them lost funding and time due to unreasonable requests and requirements. Moreover, some researchers faced ethical dilemmas while conducting their research due to the strict regulations and guidelines imposed by the regulatory bodies (Quote #18).

#### Unexpected logistical costs and obstacles

##### Research costs

Novice researchers encountered unexpectedly high research costs.

For the participants’ safety, they could not use public transportation, so the researchers had to provide higher transport refunds than initially budgeted. (Quote #19). They may have had to cut costs in other areas or seek additional funding to ensure the success of their research.

##### Expectations of money

The researchers expressed concern that many people assumed they had much money because they worked with sexual and gender minority populations. “*People believe that conducting research among these populations is for financial gain. As a result*,* we receive a barrage of requests for funding assistance*,* school fees from communities*,* and money for food to support the communities’ livelihoods*” (Quote #20). The researchers felt that this perception of research being solely for financial gain could be detrimental to the scientific community’s efforts to improve the health and well-being of these populations.

#### Criminalization of sexual and gender minorities and implications for researchers

One of the most recent critical barriers to conducting HIV research among gender minority populations has been the passage of an anti-homosexuality act. Since the enactment of this legislation, most researchers have reported a decrease in the number of participants willing to participate in their studies, the number of participants who have visited HIV treatment facilities, and the number of gender minority participants who have visited study sites for follow-up visits (Quote #21). On the other hand, researchers are concerned because the law does not specify the penalty for working with these populations, leading to uncertainty and self-doubt about whether it is currently legal or illegal to conduct research among gender minority populations (Quote #22).

### Lesson learned while conducting HIV research among gender minority populations

The third theme that emerged was the researchers’ lessons learned. Participants believed these lessons could be applied by other HIV researchers working in similar settings, particularly those working with gender minority populations for the first time. Figure 1 shows the coding tree, and supplemental Table 1 shows the narrative quotes.

#### Navigating the ethical approval and study site engagement process

##### Research regulation

Following the lengthy delays in the research process, the participants recommend beginning the ethical approval process early to account for any unexpected delays. They also suggested that researchers talk to other people who work in similar contexts. “*By talking to other HIV researchers working with similar populations*,* people can gain insights into potential challenges and ways to mitigate them*” (Quote #23). They also suggested that these researchers should know the legal status of transgender people, gender dysphoria, and transgender-specific healthcare needs.

##### Administrative clearance

In addition, participants suggested that researchers contact study sites before requesting ethical approval. “*This engagement will make obtaining administrative approval easier*,* as the sites will already be aware of the study and be more likely to grant permission due to earlier community engagement. This early engagement signifies respect and makes gaining entry*,* approval*,* and stakeholder buy-in easier.”* (Quote #24). They suggested that establishing early contact with study sites could help researchers better comprehend the local context and potential obstacles that may arise during the study, allowing for more efficient planning and implementation.

#### Diversity and sensitivity training

Participants had to undergo diversity and sensitivity training before working with people from gender minority groups. According to the participants, this training allowed them to interact with the peer trainers and learn the appropriate terminology to avoid offensive or disrespectful language, such as misgendering a trans person (Quote #25). Before beginning work with these populations, the participants suggested that future researchers who engage in similar endeavours should complete this training.

#### Leveraging networks through trust building

The study’s success was only possible through intentionally utilizing existing networks, systems, and structures. To ensure future success, the researchers emphasised the importance of community engagement, the use of social networks, and respect for pre-existing structures. They also highlighted the importance of building trust with these communities by “*acknowledging their concerns and experiences and involving them in the research process to ensure their voices and needs are prioritised*” (Quote #26).

#### Meaningful community engagement and participation

All participants emphasized the significance of involving the community in HIV research.

##### Design and implementation

Several of them learned from costly errors. They learned that what they had envisioned in their designs did not occur in real-world environments. Participants unequivocally advocated for the inclusion of gender minority populations at “*all stages of HIV research*,* from the study’s conceptualization to the protocol design to the pursuit of ethical approval*,* to the implementation of their study”* (Quote #27). They asserted that some members of the transgender and gender minority populations were more inclined to communicate with HIV researchers when their teams included peer research assistants.

##### Dissemination

Participants emphasised the importance of collaborating with members of these populations and communities while disseminating study results, particularly those relating to gender minority groups. HIV researchers who did not originate from these populations also felt it strengthened their relationships with research participants and led to more meaningful and impactful research outcomes. “*This approach also helped us to build trust and mutual respect with our participants*,* ultimately benefiting the broader community*” (Quote #28). Additionally, “*involving community members in the dissemination process can increase the relevance and applicability of research findings”* (Quote #29).

#### Reflexivity

Researchers emphasised the importance of humility and self-awareness for those intending to work with populations from gender minority groups. They recommended keeping a reflexive journal to track how their attitudes, thoughts, and perceptions of these communities evolved as their research progressed (Quote #30). This reflexivity led to a more nuanced understanding of the communities they were studying and a deeper appreciation for the complexity of social issues. Some researchers found that writing in a journal helped them deal with the stigma they and the people in their studies faced (Quote #31).

#### Ensuring safety

Safety emerged as a crucial factor in researchers’ experiences and the lessons learned while dealing with gender minority groups in Uganda.

##### Personal safety

Researchers recommended that individuals working in highly stigmatised or criminalised contexts consider their safety, as there was a genuine possibility of violence. The Ugandan parliament’s passage of an anti-homosexuality law exacerbated this stigma. Additionally, some researchers who continued their work in Uganda implemented stricter safety measures to protect themselves and their participants from potential harm (Quote #32).

##### Participants safety

The HIV researchers emphasized participant safety when working with criminalised populations such as the gender minority. They recommended that risk management plans and safety protocols be implemented before the start of any research to ensure the safety of all participants, regardless of their gender identity, expression, or sexual orientation (Quote #33).

##### Data safety

The data’s security, specifically the participants’ identity and identifiers, was also a significant source of concern. Several researchers suggested implementing a data safety and security plan before researching minority populations. This plan should cater to both paper and electronic data storage. “*Such a plan should also include measures to ensure that the data is only accessible to authorized personnel and that any breaches are reported immediately to the affected individuals and appropriate authorities*” (Quote #34).

#### Equal partnership

Participants suggested that for HIV research among gender-minority populations to be successful, community members and researchers must be equal partners. They recognize that these populations are distinct from those who typically participate in research and then disappear and that “*transgender individuals are interested in participating in the process*” (Quote #35). Future researchers must recognize that trans individuals deeply understand themselves and their experiences. Therefore, they must be involved in designing solutions to their daily challenges.

#### Giving feedback

Researchers suggested a unique dissemination and feedback session for transgender and gender-diverse communities after the study. This meeting would allow dialogue between these populations and determine whether the findings represented their population (Quote #36).

#### Knowledge and awareness of legal implications

Initially, some HIV researchers were unaware of the position of gender minority populations in the country’s legal system. They encountered questions regarding the legality of interacting with transgender individuals during the ethical approval process and when they sought administrative clearance, particularly from government facilities that did not wish to break the law (Quote #37). Therefore, they were required to seek legal counsel and familiarise themselves with the rules and penalties for working with gender minority populations.

## Discussion

This study found that HIV researchers working with gender minority populations in hostile environments encounter positive and negative experiences. While the researchers share similar experiences with those studying other key populations, the results highlight the importance of utilising existing sexual and gender minority (SGM) networks, undergoing diversity and sensitivity training, ensuring their safety and the safety of study participants, and maintaining a constant reflexive mindset [25]. Specifically, researchers recommend collaborating with these populations throughout the research process, from conception to implementation and dissemination. Additionally, other studies encourage formulating dissemination, adaptation and sustainability plans for co-created interventions [26]. This approach can help ensure that the research is culturally sensitive and relevant to the community and help researchers and participants develop a sense of trust. Involving SGM members in the research process can also result in more precise data collection and interpretation.

The shift in attitudes, preconceived notions, and misconceptions among health researchers was one of the most frequently reported positive aspects of working with gender minority populations (see Table 1, quote 1). Health professionals advise researchers to engage in internal reflection to examine their perceptions and bracket these perceptions during the study [27, 28]. Participants suggested that researchers look at trans people holistically, seeing them as more than their gender identity or sexual orientation [29, 30]. Some participants remembered making mistakes in the past, such as misgendering study participants and using language that they later discovered was offensive [31]. Researchers must acknowledge and learn from these mistakes to ensure that their research is inclusive and respectful of all individuals.

Furthermore, the involvement of the gender minority community resulted in a more extensive network for the researchers, access to study participants, and an additional list of allies. Gatekeepers included community organisations, peer counsellors, and other key stakeholders. The researchers emphasized the potential to engage ‘hidden’ transgender and non-binary communities through referrals and recommendations. However, studies advise researchers to move beyond merely involving trans individuals as a conduit to connect with other participants. Instead, they should actively engage trans people at all stages of the research process [32]. Trans people are often used as “tokens” and only valued for what they can do rather than for who they are and their expertise [26]. Consequently, cultural humility and ongoing education are essential for researchers to approach their work with care.

HIV researchers working on gender minority populations faced several challenges and raised some concerns. Among them were the unexpected stigma from coworkers, social isolation, fear for personal safety, and working in an environment where gender minorities are criminalised. In addition to the inequities patients face when seeking care, research suggests that SGM health professionals face workplace discrimination [33]. Some cisgender health professionals who work with SGM populations choose not to disclose their work due to fear of backlash from the community [34]. Similarly, some are hesitant to publish their findings for the same reason. Researchers in Malawi, where same-sex relationships are illegal, found that unconventional community engagement methods were necessary to ensure the safety of study participants and staff. They also stressed the importance of getting police and media on board with the study before starting [35].

In addition, researchers reported difficulties with budgeting and logistics due to increased spending beyond initial projections and other unanticipated costs. Other studies have also reported these costs and complications with logistics [15]. These difficulties arose because of the unique safety requirements and worries of the transgender community. The interviews were held in expensive, secure locations, and private transportation was used. Participants who felt unsafe during their scheduled interviews rescheduled. Security measures were taken to ensure the safety of the participants and maintain confidentiality. This is just one example that shows how different it is to study members of underrepresented groups and how important it is to consult with seasoned researchers in the same field and underrepresented group members [36].

Before designing or conceptualising the study, the researchers learned that it was crucial to learn about their gender minority participants and any existing laws governing their rights and participation [35]. Further, they suggested that the research process would be more likely to proceed smoothly if designed in partnership and collaboration with gender minority individuals, such as the community-based participatory research approach [37]. Vincent (2015) suggests that to have ethical recruitment and collaboration with transgender participants in academic research; researchers need to know their history [38]. This necessitates a thorough review of the literature and an understanding of the historical and current legal status of trans people in that environment [39]. For instance, ‘‘Sect. 381 of the Ugandan Penal Code act, for example, defines impersonation as a person falsely representing himself or herself intending to defraud another,‘ [40] but the same act is silent on trans people. As a result, researchers must investigate the application of this law to trans people and whether it will discriminate against or persecute them [41]. Furthermore, it is critical to consider social and cultural attitudes towards trans people and how they may impact their interactions with the criminal justice system [42].

Protecting researchers, participants, and data is paramount. Several studies document the need for safety when working with trans populations [43]. In bioethics, nonmaleficence entails a commitment to minimise harm to participants [44, 45]. This harm minimisation is achievable through informed consent procedures, confidentiality measures, and other ethical guidelines. The *Respect*, P*rotect*, and *Fulfill* human rights framework asserts that protecting the rights of vulnerable people also involves anticipating and developing mechanisms to prevent the violation of their human rights or social harm by others [46]. Harm in research may be perceived as perpetuating stigma and discrimination, “outing” due to data presentation, and any form of violence [38]. Avoiding harm means that HIV researchers must ensure that the rights of research participants and staff are not violated due to their participation in research.

Gender diversity and sensitivity training may benefit researchers new to working with gender minority populations. The researchers’ willingness to go above and beyond to learn about transgender people demonstrates their respect. Several studies found that this training improved the attitudes of research teams and their provision of SGM culturally responsive care [2]. Reback and colleagues recommend that new staff observe senior staff in the field and participate in a series of mock outreach encounters to practise dealing with difficult situations [47]. Nurses who received this training reported improved attitudes and empathy for vulnerable and key populations [48]. Empathy fosters a supportive and non-judgmental environment, significantly impacting patient outcomes. Training health workers in empathetic communication enhances their ability to address patients’ needs effectively, build trust, and reduce stigma, ultimately leading to improved adherence to treatment and overall care quality [49].

The positive findings also highlight the importance of ongoing education and training for health researchers. Training on transgender health care is critical for health practitioners and researchers to ensure equitable and effective care for transgender individuals [50]. Such training equips professionals with the knowledge and skills necessary to address the unique medical and psychological needs of transgender patients, including understanding gender dysphoria, providing gender-affirming care, and navigating the complexities of legal and ethical considerations [51]. For researchers, comprehensive training in transgender health issues enhances the design and implementation of studies, ensures culturally competent data collection, and fosters more inclusive research practices. By integrating this specialized training into professional development, the healthcare system can improve patient outcomes, reduce health disparities, and advance respectful and informed research practices.

Several reports have urged researchers to include transgender women, men, and non-binary people who are at risk of or living with HIV in their studies, not just as peer recruiters but as methodological and scientific experts [52, 53]. The underlying theory is that when the research knowledge base is biased in terms of gender or other types of diversity, it diminishes the trustworthiness of the research and its benefit to society [54]. It becomes questionable whether the research used the appropriate questions and methods to elicit relevant findings for improving transgender health [55]. Each partner makes a unique contribution and shares some responsibility for understanding the social and cultural dynamics of the phenomenon being studied [56]. However, it will be easier to form partnerships depending on how organised social movements and community groups are. For instance, Dias and colleagues reported that creating partnerships with men who have sex with men was significantly more straightforward than with sex workers [25]. Therefore, it is crucial to ensure that HIV research is conducted inclusively and diversely, considering trans individuals’ perspectives and experiences.

### Study limitations and recommendations for further research

The qualitative nature of this research, while offering in-depth insights, relies on subjective experiences that may not generalize to all criminalized gender minorities in Uganda. Additionally, legal and social barriers limited participant access, affecting the depth of the information gathered.

Future research should use mixed methods approaches to combine qualitative and quantitative data to address these issues for a more comprehensive understanding. Innovative strategies to reach hard-to-access populations and navigate legal and social barriers are essential for obtaining representative data. Longitudinal studies could also provide valuable insights into the long-term effects of HIV research and interventions, informing more effective and inclusive health policies.

## Conclusions

This study highlights both the significant challenges and invaluable lessons learned while conducting HIV research among criminalized gender minority populations in Uganda. The research underscores the importance of respecting cultural diversity, expanding networks, and addressing misconceptions to build trust and enhance the effectiveness of HIV interventions. However, the study also reveals the severe stigma, discrimination, and safety concerns that both participants and researchers face, emphasizing the need for robust ethical guidelines and protective measures.

Moving forward, researchers must leverage networks, engage meaningfully with communities, and practice reflexivity to address their biases. Furthermore, ensuring the safety of both participants and data must be a top priority. Despite the obstacles, this work provides a foundation for future inclusive, sensitive, and effective research approaches, with the goal of improving healthcare access and outcomes for gender minorities in Uganda.

Future research should continue to build on these insights, addressing the identified challenges and expanding the scope to include more extensive, diverse populations. Additionally, collaboration with experienced researchers and community members will be vital to overcoming barriers and ensuring that the research advances knowledge and contributes to the well-being and rights of marginalized populations.

## Data Availability

No datasets were generated or analysed during the current study.

## Declarations

### Patient and public involvement

No patients or members of the public were involved in the planning, execution, reporting, or dissemination of this study.

## Abbreviations

GBV: Gender based violence
HIV: Human Immunodeficiency Virus
IPA: Interpretative phenomenological analysis
KII: Key informant interview
LGBTQ: Lesbian, gay, bisexual, transgender, queer
SGM: Sexual and gender minority
WHO: World Health Organization
